# War stories

**DOI:** 10.1017/S1478951524002013

**Published:** 2025-04-07

**Authors:** James Seymour Huntley

**Affiliations:** Department of Pediatric Orthopedics, University of Utah, Salt Lake City, UT, US

Hospital visiting-hours:
“*Hello John, heard you were*
*having a spot of bother*.”
Old men, knowing how to talk,
in the dusk return:
in youth they moved the bodies
of friends into the sun.
Their tender violent brotherhood
was all, gave all -
packed like jewels -
I envy them their friends,
their stories, their loves,
their war.

